# Gill Damage to Atlantic Salmon (*Salmo salar*) Caused by the Common Jellyfish (*Aurelia aurita*) under Experimental Challenge

**DOI:** 10.1371/journal.pone.0018529

**Published:** 2011-04-07

**Authors:** Emily J. Baxter, Michael M. Sturt, Neil M. Ruane, Thomas K. Doyle, Rob McAllen, Luke Harman, Hamish D. Rodger

**Affiliations:** 1 Coastal and Marine Research Centre, Environmental Research Institute, University College Cork, Cork, Ireland; 2 Department of Zoology, Ecology and Plant Science, School of Biological, Earth and Environmental Sciences, University College Cork, Cork, Ireland; 3 Aquaculture and Fisheries Development Centre, School of Biological, Earth and Environmental Sciences, University College Cork, Cork, Ireland; 4 Fish Health Unit, Marine Institute, Co. Galway, Ireland; 5 Vet-Aqua International, Co. Galway, Ireland; Biodiversity Insitute of Ontario - University of Guelph, Canada

## Abstract

**Background:**

Over recent decades jellyfish have caused fish kill events and recurrent gill problems in marine-farmed salmonids. Common jellyfish (*Aurelia* spp.) are among the most cosmopolitan jellyfish species in the oceans, with populations increasing in many coastal areas. The negative interaction between jellyfish and fish in aquaculture remains a poorly studied area of science. Thus, a recent fish mortality event in Ireland, involving *Aurelia aurita*, spurred an investigation into the effects of this jellyfish on marine-farmed salmon.

**Methodology/Principal Findings:**

To address the *in vivo* impact of the common jellyfish (*A. aurita*) on salmonids, we exposed Atlantic salmon (*Salmo salar*) smolts to macerated *A. aurita* for 10 hrs under experimental challenge. Gill tissues of control and experimental treatment groups were scored with a system that rated the damage between 0 and 21 using a range of primary and secondary parameters. Our results revealed that *A. aurita* rapidly and extensively damaged the gills of *S. salar*, with the pathogenesis of the disorder progressing even after the jellyfish were removed. After only 2 hrs of exposure, significant multi-focal damage to gill tissues was apparent. The nature and extent of the damage increased up to 48 hrs from the start of the challenge. Although the gills remained extensively damaged at 3 wks from the start of the challenge trial, shortening of the gill lamellae and organisation of the cells indicated an attempt to repair the damage suffered.

**Conclusions:**

Our findings clearly demonstrate that *A. aurita* can cause severe gill problems in marine-farmed fish. With aquaculture predicted to expand worldwide and evidence suggesting that jellyfish populations are increasing in some areas, this threat to aquaculture is of rising concern as significant losses due to jellyfish could be expected to increase in the future.

## Introduction

With the intensified use of the marine environment and evidence suggesting that jellyfish populations are increasing in some areas, it is not surprising that reports of jellyfish blooms interfering with anthropogenic activities (including aquaculture) are rising [1]. Over recent decades, jellyfish blooms of species such as the siphonophore *Muggiaea atlantica*, the small, oceanic hydromedusa *Solmaris corona,* and the oceanic scyphomedusa *Pelagia noctiluca*, have caused the death of hundreds of thousands of farmed salmonids in a number of regions throughout Europe [2], [3], [4]. Jellyfish blooms that aggregate around aquaculture farms may either pass through the mesh of aquaculture cages in whole or in part (when blooms degrade or tentacles extend into the cages), depending on the size of the individuals [2]. Damage to fish in aquaculture may therefore be direct, through stinging of the skin or gills (if small individuals or loose nematocysts are inhaled), or indirect, through de-oxygenation of the surrounding water [2].

Gill disorders have become one of the most serious causes of mortality in marine farmed salmon in Ireland alone, with average losses of 12% per annum (range: 1 to 79%) being experienced throughout the industry [5], [6]. The aetiology of gill disorders is complicated with many possible causative agents including jellyfish, phytoplankton, bacteria, viruses and parasites, with damage from any one of these agents often leading to increased respiratory and osmoregulatory stress, and subsequently death [5], [7]. Recent research has also implicated jellyfish as vectors of bacterial disease; whereby physical damage to the gills caused by nematocysts may be exacerbated with the introduction of bacterial pathogens, such as *Tenacibaculum maritimum*, carried by the jellyfish [8], [9]. However, information in the literature about interactions between jellyfish and fish in aquaculture remains limited, not only in terms of the number of reported incidences but also to the species implicated. Furthermore, insufficient data exist about the species-specific pathogenesis and pathological damage caused by many jellyfish species that commonly occur around sites of aquaculture.

The common jellyfish, of the genus *Aurelia*, is one of the most cosmopolitan jellyfish in our oceans, with populations increasing in many coastal areas worldwide; much to the detriment of coastal industries such as fishing and power plant operations [10]. The sting of *Aurelia* spp. is considered quite benign to human skin inflicting only a very mild sting in thin-skinned regions of the body [11]. However, there have been a number of recorded incidences of *Aurelia* spp. interfering with aquaculture operations [2], [12], [13], [14]. A recent, fish kill event at a commercial Atlantic salmon farm off the west coast of Ireland, in the summer of 2010, prompted an investigation into the impacts of this common and abundant species on marine-farmed fish. The aim of the current study was to explore the problem of gill damage in Atlantic salmon (*Salmo salar*) caused by the common jellyfish (*A*. *aurita*). To examine detailed histopathological changes in the gill tissues of experimental treatment and control fish, an *in vivo* experimental challenge trial was conducted with samples taken over a time series of up to 3 wks.

## Results

### Gross pathology

In response to 10 hrs exposure to macerated *A. aurita* tissue, some fish in the experimental treatment groups displayed persistent gross pathological changes of the gills over the course of the study. This presented as focal patches of haemorrhage or necrosis of the lamellae after 2 hrs of exposure. No other lesions were visible grossly on external or internal examination of the fish at any stage.

### Histopathology and gill scores

Throughout the challenge trial, the control groups had healthy-looking gills with background levels of epithelial hyperplasia (cell proliferation) in small areas that were considered standard for farmed fish [H. D. Rodger, pers. obs.]. The gill scores of the control groups ranged from 0–5 over the course of the trials, with the fish showing no significant histopathology. Some fish showed signs of minor gill pathology; however, this was considered to be indicative of background damage. No parasites or bacteria (e.g. *Tenacibaculum* sp.) were observed on the gills of any fish over the duration of the experiment.

**Figure 1:**
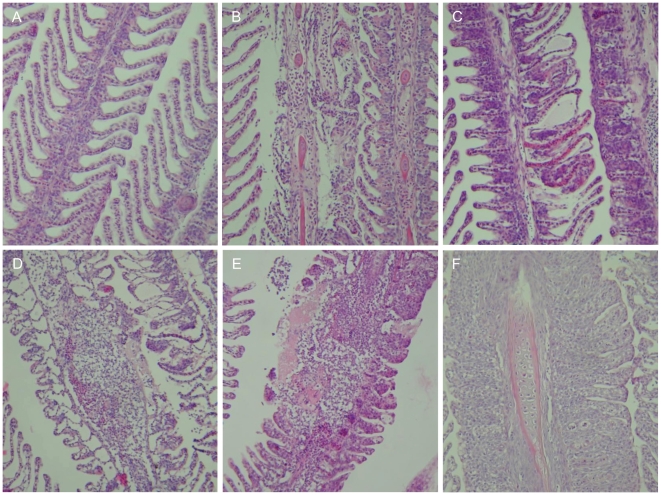
Photographic time series of gill lesions in fish exposed to *Aurelia aurita* under experimental challenge. Times expressed in hours from the start of the experiment. A: Healthy gills from control group (0 hr). B–F: Gills from experimental treatment groups. B: 2 hrs. C: 6 hrs. D: 24 hrs. E: 48 hrs. F: 3 wks. Using haematoxylin and eosin at 200× magnification.

**Figure 2:**
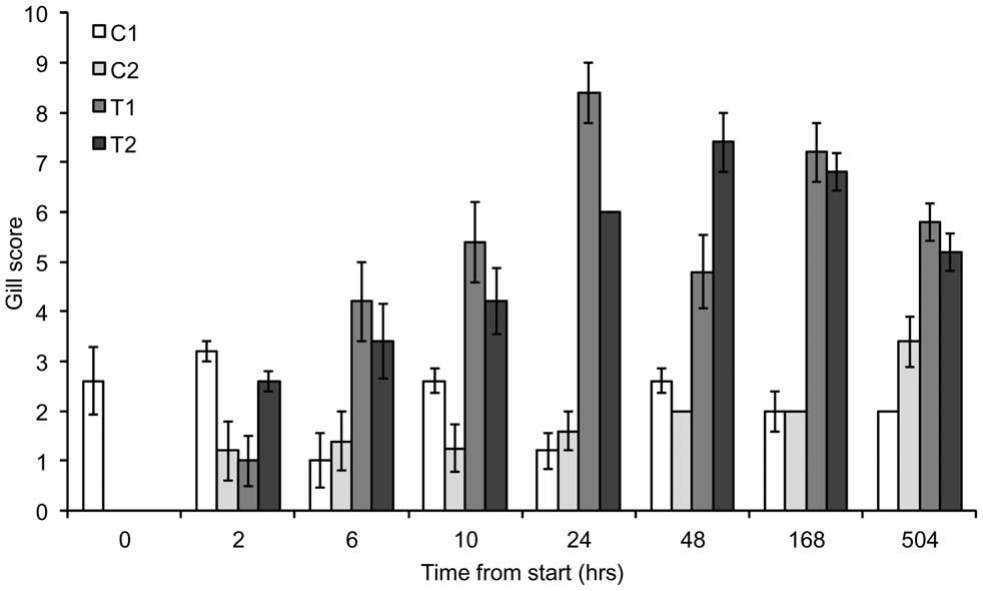
Gill scores of control and test groups with time from the start of the experiment. Gill scores for Control group 1 (C1), Control group 2 (C2), Test group 1 (T1) and Test group 2 (T2). All values are means ± 1 S.E. (n = 5).

The gill lesions observed in the fish exposed to the jellyfish significantly worsened over the course of the challenge trial with a peak in gill damage 48 hrs after the start of the challenge (Figs. 1 and 2). At 3 wks (504 hrs) from the start of the experiment there were signs of early stage repair in the gill tissues. The gill scores for the experimental treatment groups ranged from 3–9 over the entire experiment, with most fish displaying moderate lesions considered to be of clinical significance (Fig. 1).

The clinical signs of gill disease for each sampling point detailed in hrs from the start of the experiment were: (a) 2 hrs–lamellae with multi-focal areas of epithelial sloughing, necrosis and haemorrhage; (b) 6 hrs–increased lamellar epithelial sloughing, oedema and necrosis. Focal haemorrhages and cellular hypertrophy were also associated with the damaged areas; (c) 10 hrs–multi-focal areas of haemorrhage, epithelial stripping (large areas where the epithelium was missing entirely), necrosis and oedema with obvious inflammation of the gill filaments as evidenced by the presence of granulocytes; (d) 24 hrs–all lesions detailed above were more extensive with significant necrotic and inflammatory areas and the appearance of ‘ghost’ cells (denucleated cells); (e) 48 hrs–the severity of the gill lesions increased once again with large areas of epithelial stripping, haemorrhage and lysis of erythrocytes; (f) 168 hrs (1 wk)–secondary lamellae fused throughout their length with significant hyperplasia and multiple small blood clots; (g) 504 hrs (3 wks)–extensive hyperplasia and fusion of lamellae remained; however, there was a reduction in the extent of the damage with few obvious signs of necrosis, sloughing or oedema. Shortening of the lamellae and organisation of the cells indicated an attempt to repair the damage suffered.

There was a highly significant difference in gill scores between the experimental treatment and the control groups and no significant difference within group replicates (Table 1). There was also a significant interaction with time; gill scores from 24 to 504 hrs (3 wks) from the start of the experiment were significantly higher than the first 3 sampling periods (Table 1).

**Table 1:** Two-way ANOVA for gill scores across groups and sampling times.

Source of variation	df	F	*p*	Significant pairwise comparisons
Group	3	107.97	<0.001	T1 & T2 > C1 & C2*
Time	6	8.77	<0.001	0 hrs < 68 hrs*
				2 hrs < 24, 48 & 168 hrs* 6 hrs < 24, 48, 168, 504 hrs*
				10 hrs < 168 hrs*
Group*Time	18	5.49	<0.001	-
Error	112			
Total	141			

Groups: T1  =  test group 1, T2  =  test group 2, C1  =  control group 1, C2  =  control group 2; Sampling times are in hours (hrs) after the start of the experiment. *Indicates significance at *p* <0.001.

## Discussion

Several species of jellyfish have been observed to cause both large scale fish kill events and the more chronic problem of gill damage in marine-farmed fish. The potential interaction of the widespread and abundant jellyfish *A. aurita* with finfish aquaculture may have been previously underestimated and understudied. Recently, *A. aurita* was implicated in a severe case of gill damage and fish mortality on the west coast of Ireland [H. D. Rodger, pers. comm.], an incident which drove our investigation into the potential pathogenesis of gill disorders caused by this jellyfish. Importantly, our study represents the first experimental challenge trial undertaken with fish in culture and jellyfish.

In our experiments, Atlantic salmon showed distinct pathological changes to their gills with the loss of epithelial cells, focal haemorrhages and the onset of necrosis after only 2 hrs of exposure to *A. aurita* tissue. Importantly, the gill damage caused by *A. aurita* over the course of the trial significantly increased with time, even after the jellyfish had been removed. The fish also displayed a delayed inflammatory response that was obvious at 24 hrs after exposure as granulocytes became concentrated in the filaments. These results imply that even short-term exposure to jellyfish (for example, over a tidal cycle) could result in significant gill damage in marine-farmed fish; with the damage having the potential to progress in extent and severity even if no jellyfish are present. The exposed fish in the present study would have experienced severe respiratory and osmoregulatory problems throughout the course of the challenge trial, which may have compromised their survival under natural conditions. Interestingly, there were no mortalities of exposed fish and after 3 wks (504 hrs) from the start of the trial recovery appeared to have been initiated. However, in the natural environment without UV sterilisation and filtration systems, secondary bacterial infections, which are known to exacerbate gill damage [5], may have seriously impeded or prevented recovery and may have ultimately resulted in death.

Recently, an *in vitro* approach was used to demonstrate the cytotoxicity of cnidocyst extracts from *A*. *aurita* on rainbow trout gill assays at a cellular level [15]. Our data concur with these findings and additionally provide a critical, novel *in vivo* approach to assess the direct impact of an individual jellyfish species on farmed fish. *Aurelia* spp. medusae form particularly dense aggregations in many regions worldwide [16], [17], an attribute that may be enhanced by their swimming behaviour in response to certain hydrodynamic conditions [18]. A significant increase in the abundance, frequency of occurrence and the distribution of *Aurelia* spp. over recent decades has also been noted in some locations [19]. Although speculation remains as to whether jellyfish populations are increasing in response to climate change, shifting climate cycles may alter the distribution and seasonal occurrence of temperate-boreal jellyfish species such as *Aurelia* spp. [20].

Over the last decade, the average yearly growth in the production of marine fishes was 11.9% worldwide. Although the rate of expansion may slow over the coming years, it is predicted that the aquaculture industry will continue to grow alongside an increased demand for fish and a decline in fisheries landings [21]. Therefore, the likelihood of detrimental interactions between *Aurelia* spp. and aquaculture operations can be expected to increase in the future. Furthermore, previous observations have shown that aquaculture pontoons may act to enhance *A. aurita* populations, possibly by providing a substrate for polyp settlement and growth as well as restricting water flow around the pontoon structures aiding medusa retention [22]. Consequently, the increased use of the marine environment for aquaculture may inadvertently promote jellyfish blooms and may subsequently have severe implications for fish health and survival throughout the bloom periods. Future studies investigating ecologically sound mitigation methods to prevent the biofouling of jellyfish polyps on aquaculture structures, as well as methods to prevent jellyfish material from entering the fish cages are essential if the significant economic losses and the impact on finfish health in aquaculture are to be limited.

The impacts of jellyfish blooms on finfish in aquaculture are not exclusive to salmon production, and are likely to occur in all areas where Aurelia spp. and other jellyfish are common. Our data have global relevance, as jellyfish have affected or may potentially affect highly productive aquaculture operations in regions such as Asia, north-western Europe, Australia and South America [2], [3], [13], [22]. In summary, our study clearly demonstrates that *A. aurita* is as an agent of severe gill disease in marine-farmed salmon. The findings presented here improve our understanding of the potential threat of this cosmopolitan jellyfish to finfish aquaculture, as well as our knowledge of the nature and extent of the damage caused which will be applicable to field investigations.

## Materials and Methods

### Ethics statement

This study was carried out in strict accordance with the recommendations for the care and use of Laboratory Animals in Science and Training (Ireland) and the experimental protocol was conducted under the regulations of the Cruelty to Animals Act 1876, as amended by European Communities Regulations 2002 and 2005, approved by the Department of Health and Children, Ireland (Licence number: B100/4280). The protocol was approved by the University College Cork Animal Experimentation Ethics Committee (Review number: 2009/#41). All sampling was performed post-mortem on animals that were euthanized with a lethal dose of MS-222 (tricaine methanesulfonate). All efforts were made to minimise suffering. The facilities at the Aquaculture and Fisheries Development Centre were subject to inspection and approved as suitable premises for experimental procedures by the Department of Agriculture, Fisheries and Food, Ireland.

### Fish maintenance

Hatchery-raised (S1, 1 year old) Atlantic salmon smolts were obtained from the hatchery at the Marine Institute, Furnace, Newport, County Mayo, Ireland and seawater adapted on arrival at the Aquaculture and Fisheries Development Centre, University College Cork, Ireland. The experimental setup comprised of 44 fish in each control (x 2) and experimental (x 2) treatment group. The fish had an initial average weight of 65.1±19.6 g (mean ± 1 S.D., n = 65) and were kept at a stocking density of 9.5 kg/m^3^. All groups were maintained in 300 L of flowing seawater (salinity, 33‰) at 11±1°C with supplemented air to keep dissolved oxygen at 7.5 mg/L or higher. The photoperiod was maintained on a 12 hr light:12 hr dark cycle and the fish were fed a standard commercial pellet diet (Skretting: Atlantic smolt) throughout the day by automatic feeders.

### Experimental setup

The challenge specimens of the common jellyfish *A. aurita*, were collected 48 hrs prior to the challenge start time from Glengarriff Harbour in Glengarriff Bay, south-west Ireland (51°44′56 N, 09°32′33 W). The jellyfish were collected by hand from the harbour, placed in buckets and kept cool with ice blocks until returned to the laboratory. In the laboratory, the jellyfish were transferred to a clean, isolated seawater tank without food until the start of the trials. Immediately prior to the challenge trials, the jellyfish were weighed out into two buckets of equal biomass (1.8 kg, ∼20 individuals). In order to simulate the exposure of marine-farmed salmon to jellyfish, which must have first passed through the cage mesh, the jellyfish were macerated into pieces a few centimetres in diameter.

The outflows of all tanks containing the experimental and control fish groups were covered with a 1-mm stainless steel mesh. This was undertaken in order to prevent large pieces of jellyfish leaving the experimental tanks and to deliver the same environment in the control tanks. The filtration system in the re-circulation unit was fitted with 5-µm mesh mechanical filter bags (and changed regularly) to prevent gelatinous material or free nematocysts from entering the re-circulation system. The fish were also kept off feed for 24 hrs prior to the start of the challenge.

### Sampling

Prior to the challenge (time 0 hr), 5 fish were sampled randomly across the tanks. The challenge began when the macerated jellyfish were added to the two experimental treatment tanks. The fish in the experimental groups were exposed to the macerated *A. aurita* for 10 hrs before all gelatinous material was removed. Five fish were sampled from each tank at 2, 6, 10, 24, 48, 168 (1 wk) and 504 (3 wks) hrs from the start of the challenge. Fish were sampled randomly one tank at a time with buckets and nets being isolated between control and experimental treatment tanks. Fish were placed into 10 L buckets containing a lethal dose of anaesthetic (MS-222 (tricaine methanesulfonate): 100 mg/L) before their length and weight were measured and tissue samples taken.

The fish and gills were examined for gross pathology and then the second gill arch on the left-hand side was excised from each fish. Gills were immediately fixed in 10% neutral-buffered formalin for histological analysis. Tissues were then paraffin embedded and cut into 5-µm sections and stained with haematoxylin and eosin. Samples were then scanned microscopically at 40×, 100× and 400× magnifications.

### Gill scores

A semi-quantitative scoring system, developed by Mitchell *et al*. [In prep], was used for histopathological examination of the gills. The scoring system combines scores for primary and secondary criteria. The score for the primary parameters (ranging from 0–3 for each gill analysed) is based on the presence, severity and extent of: epithelial hyperplasia (increased cell production), lamellar fusion and cellular anomalies (including degeneration, necrosis and sloughing). An additional score of 1 is added accordingly for the presence (not severity) of each of the following secondary parameters: hypertrophy (enlarged cells), inflammation, oedema, eosinophilic granular cells, circulatory damage (e.g. haemorrhage, telangiectesias (dilated blood vessels), congestion, and the presence of bacterial (e.g. epitheliocystis, *Tenacibaculum* spp.) and parasitic pathogens (e.g. *Costia, Neoparmoeba, Trichodina*).

The score can thus range from 0 to 21 and be interpreted as follows: 0–3  =  no significant pathology, 4–6  =  mild gill pathology of minor clinical significance, 7–9  =  moderate gill pathology of clinical significance, ≥10  =  severe gill pathology of high clinical significance.

### Statistical analysis

A two-way analysis of variance (ANOVA) was conducted to assess the interaction between gill score in relation to **group** (2 experimental treatment and 2 control groups) and **time** from the start of the experiment. Normality and homoscadacity were tested for prior to analysis using box-plot visualisation and Levene's test respectively. Significant ANOVA results were investigated post-hoc using Tukey's pairwise comparisons.
